# Functional characterization of KS-type dehydrin ZmDHN13 and its related conserved domains under oxidative stress

**DOI:** 10.1038/s41598-017-07852-y

**Published:** 2017-08-04

**Authors:** Yang Liu, Li Wang, Tianpeng Zhang, Xinghong Yang, Dequan Li

**Affiliations:** State Key Laboratory of Crop Biology, Shandong Key Laboratory of Crop Biology, College of Life Sciences, Shandong Agricultural University, Tai’an, 271018 Shandong China

## Abstract

Dehydrins belong to the group 2 family LEA (Late Embryogenesis Abundant) proteins, which are up-regulated in most plants during cold and drought stress. According to the number and order of the Y-, S- and K-segments, dehydrins are classified into five subclasses: YnSKn, YnKn, SKn, Kn and KnS. Here, the maize (*Zea mays* L.) KS-type dehydrin gene, *ZmDHN13*, was identified and later characterized. Expression profiling demonstrated that *ZmDHN13* was constitutively expressed, but its expression was also altered by high osmosis, low temperature, oxidative stress and abscisic acid (ABA). Furthermore, the roles of the three conserved segments in phosphorylation, localization, binding metal ions and physiological functions were explored. ZmDHN13 was mainly localized in the nucleus, depending on phosphorylation status. Additional studies indicated that ZmDHN13 could be phosphorylated by CKII (casein kinase II), when the NLS (nuclear localization signal) segment and the S-segment were core sequences. The overexpression of *ZmDHN13* enhanced transgenic tobacco tolerance to oxidative stress, and the three conserved segments exhibited a cooperative effect in response to environmental stresses *in vivo*.

## Introduction

Dehydrins are characteristically rich in charged polar amino acids but lack cysteine and tryptophan^1^. Dehydrins have conserved lysine-rich motifs called K-segments (EKKGIMDKIKEKLPG), which most likely form an amphiphilic helix that can interact with membranes or proteins to modulate their phase properties and conformational transitions^2, 3^. The following are other typical conserved domains: the S-segment (a chain of Ser residues), whose phosphorylation status can influence the proteins that are transported to nucleus^4–6^, and the Y-segment (T/VDEYGNP), which is located near the N-terminus.The number and order of the K-, Y- and S-segments define the different dehydrins subclasses: YnSKn, YnKn, SKn, Kn and KnS.

Disordered structure is a common biochemical feature of dehydrins, but the K-segment, which can form an amphipathic α-helices in the presence of helical inducers, is an essential unit relevant to the function of DHNs in response to dehydration-affiliated stresses^7–10^. Under stress conditions, α-helix can stabilize membranes and proteins by protein-protein and protein-lipid interactions^11–14^.

Dehydrins can contribute to membrane and protein stabilization against environmental stresses. The maize protein RAB17 can bind to lipid vesicles that aid in membrane stabilization during stress, and the K-segment plays an important role in the interaction with PA (phosphatidic acid; anionic)^12, 14^. The lipid interaction of Lti30 *in vitro* is regulated by phosphorylation by protein kinase C, a pH-dependent His on/off switch, and the reversal of membrane binding by proteolytic digestion^15^.

Dehydrin proteins are localized in several cellular compartments, including the cytoplasm, plasma membrane^16^, nucleus, mitochondria^17, 18^, vacuolar membranes^19^ and chloroplasts^20^, of which the cytoplasm and nucleus are the main compartments^2, 4, 21, 22^. Although there are many reports of the location of dehydrins, the precise dehydrin transport mechanism is still unknown. Phosphorylation plays an important role in plant signal transduction and stress responses. The localization in the nucleus of the maize dehydrin RAB17 depends on the phosphorylation of the S-segment^23, 24^, but the K_6_-type dehydrin WCS120, which does not possess the S-segment, also localizes to the cytoplasm and nucleus; therefore, the NLS-segment present in the proteins may play an important role in the nuclear import of proteins^25, 26^.

The K-, S- and NLS-segments of KS-type dehydrin are different compared with those of other types of dehydrins. In contrast to other families of dehydrins, the K-segment of KS-type dehydrins begins with the motif (H/Q)KEG rather than EKKG, indicating that there might be a different functional mechanism for the KS-type dehydrins. Here, we explored the transcript profile and the roles of the three conserved segments in phosphorylation, localization, metal ion binding and physiological functions. This study revealed that KS-type dehydrin may be involved in various pathways and that the three conserved segments of ZmDHN13 exhibit a cooperative effect in response to environmental stresses *in vivo*.

## Methods

### Plant material and growth conditions

Maize (*Zea mays* L. cv Zhengdan 958), tobacco (*Nicotiana benthamiana*) and *Arabidopsis thaliana* were used in this study. Maize was grown in Hoagland’s solution (pH 6.0) under greenhouse conditions at 26/22 °C (day /night) with a photosynthetically active radiation of 200 μmol m^−2^ s^−1^ and a photoperiod of 16/8 h (day/night) for 2 weeks^29^. Tobacco was grown in soil in a light-emitted culture box under a photoperiod of 16/8 h (day/night) and light supplementation of 200 μmol m^−2^ s^−1^ at 25 °C. For the PEG6000, ABA and H_2_O_2_ treatments, maize and tobacco plants were watered daily unless otherwise indicated.

### Amplification and sequence analysis of ZmDHN13

Total RNA was extracted using the RNeasy Plant Mini Kit (Tiangen, China). First strand cDNAs were synthesized using the First Strand cDNA Synthesis Kit (Fermentas, USA). The whole coding sequence of *ZmDHN13* was amplified with primers (forward GGATCCAGAGAAGTAGCCACAAGCATG, *Bam*H I site underlined; reverse GAGCTCACAACAATCTTGGCGAGT, *Sac*I site underlined). The PCR product was cloned into the PMD18-T vector and sequenced. The prediction of the disorder of ZmDHN13 was carried out using IUPred (http://iupred.enzim.hu/). The hydropathy profiles of the putative proteins were constructed in accordance with the ProtScale method (http://expasy.org/tools/protscale.html). Multiple sequence alignment was carried out using the ClustalW 1.81 program (http://clustalw. genome.jp/). Statistical analyses were performed using the software programs SigmaPlot11.0 and SPSS13.0.

### Transcript accumulation analysis of *ZmDHN13*

Two-week-old maize plants were treated with 100 μM ABA, 20% PEG6000 (w/v), 20 μM H_2_O_2_, low temperature (4 °C) or water (control). The maize tissues collected from the variously treated plants at specific time points were immediately frozen in liquid nitrogen and stored at −80 °C. First-strand cDNA synthesis was performed as described above. *ZmDHN13* was amplified in the qRT-PCR reactions using primers (forward CGCATAGCATTCTCTTCC and reverse CGCTCCTGGATCTTGTC) and SYBR Green qRT-PCR SuperMix (TransGene, China). The maize actin gene (NM_001156990.1) was amplified using primers (forward CCACGAGACCACCTACAACT and reverse CCTTTCTGGAGGAGCAAC) along with the *ZmDHN13* gene to allow gene expression normalization and subsequent quantification.

### Cloning of different segment deletion genes

To generate a mutant missing the S-segment (*ZmDHN13ΔS*), a pair of primers (forward GGATCCAGAGAAGTAGCCACAAGCATG, *Bam*H I site underlined; reverse GAGCTCTCAGTGTCCGTCACCATCAC, *Sac* I site underlined) was used. Other mutants were generated as described in Supplementary Method 1.

### Protein expression and purification

Recombinant proteins were obtained using the pET30a *Escherichia coli* (BL21 DE3) expression system. The proteins were purified using a Ni–NTA spin column (Novagen), and the 6 × His tag was then removed by on-column thrombin (GE healthcare) digestion following the manufacturer’s instructions. The purity was tested by SDS-PAGE. Protein quantification was accomplished using the bicinchoninic acid assay. The pure proteins were then exchanged into a low-medium salt buffer (20 mM Tris-HCl, 100 mM NaCl, pH 8) using the HiPrep Desalting column (GE Healthcare)^27, 28^.

### Phosphorylation analyses of ZmDHN13

The phosphorylation of the recombinant proteins ZmDHN13, ZmDHN13ΔK, ZmDHN13ΔS and ZmDHN13ΔNLS was analyzed using casein kinase (CKII, New England). The methods were performed as previously described^30^. The reaction mixtures (100 U CKII, 200 μM ATP, 2 μg recombinant proteins) were incubated at 30 °C for 2 h. The samples were analyzed by SDS-PAGE. The phosphoproteins were detected using the Pro-Q Diamond Phosphoprotein Gel Staining Kit (Invitrogen) according to the manufacturer’s instructions.

### Tobacco transformation

The correct coding regions of ZmDHN13, ZmDHN13ΔK, ZmDHN13ΔS and ZmDHN13ΔNLS were cloned into the binary pBI121 expression vector under the control of the cauliflower mosaic virus (CaMV) 35S promoter. The constructs were then transformed into *Agrobacterium tumefaciens* strain LBA4404, which was then transformed into tobacco plants by the leaf disc transformation method^31^.

### Subcellular localization of each fusion protein

For the *ZmDHN13*-GFP, *ZmDHN13ΔK*-GFP and *ZmDHN13ΔNLS*-GFP fusion constructs, each frame was amplified with primers (forward TCTAGAAGAGAAGTAGCCACAAGCATG, *Xba*I site underlined; reverse GGTACCGTCGCTGTCGCTGCTGCT, *Kpn*I site underlined) from the relative plasmids. *ZmDHN13ΔS* was cloned by primers (forward TCTAGAAGAGAAGTAGCCACAAGCATG *Xba*I site underlined; reverse GGTACCGTGTCCGTCACCATCACCAT *Kpn*I site underlined) from the relative plasmids. All fragments were cloned into the pBI121-GFP vector, generating a C-terminal fusion with the green fluorescence protein (GFP) gene controlled by the CaMV 35S promoter. The constructs were transformed into *Agrobacterium tumefaciens* strain LBA4404, which was then transformed into tobacco plants by the leaf disc transformation method^31^.

### Measurements of physiological parameters

Six-week-old transgenic lines and the control line were treated with 70 μM CuCl_2_ or 20 mM H_2_O_2_ for the indicated time. Relative electrolytic leakage was performed as previously described^32^. MDA and superoxide radical (O_2_^−^) concentrations were measured as described in Supplementary Methods 2 and 3. The experiments were repeated at least three times.

### Histochemical detection of O_2_^−^

Leaves were infiltrated with 0.5 mg ml^−1^ nitroblue tetrazolium (NBT) for 20 h in the dark to detect O_2_^−^. Then, the seedlings were decolorized by boiling in ethanol (96%) for 10 min. After cooling, the leaves were extracted at room temperature with fresh ethanol and imaged using a stereomicroscope.

## Results

### Isolation and sequence analysis of *ZmDHN13*

**Figure 1:**
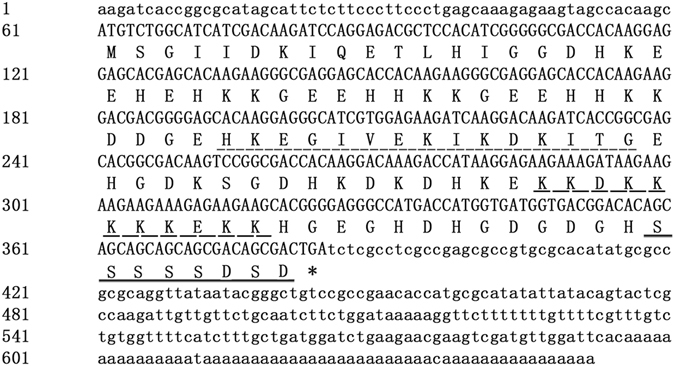
Sequence analysis of the ZmDHN13 protein. Nucleotide sequence of *ZmDHN13* cDNA together with its predicted amino acid sequence. The putative different conserved segments are indicated as the K-segment (short-dashed line), the NLS segment (long-dashed line) and the S-segment (solid line).

**Figure 2:**
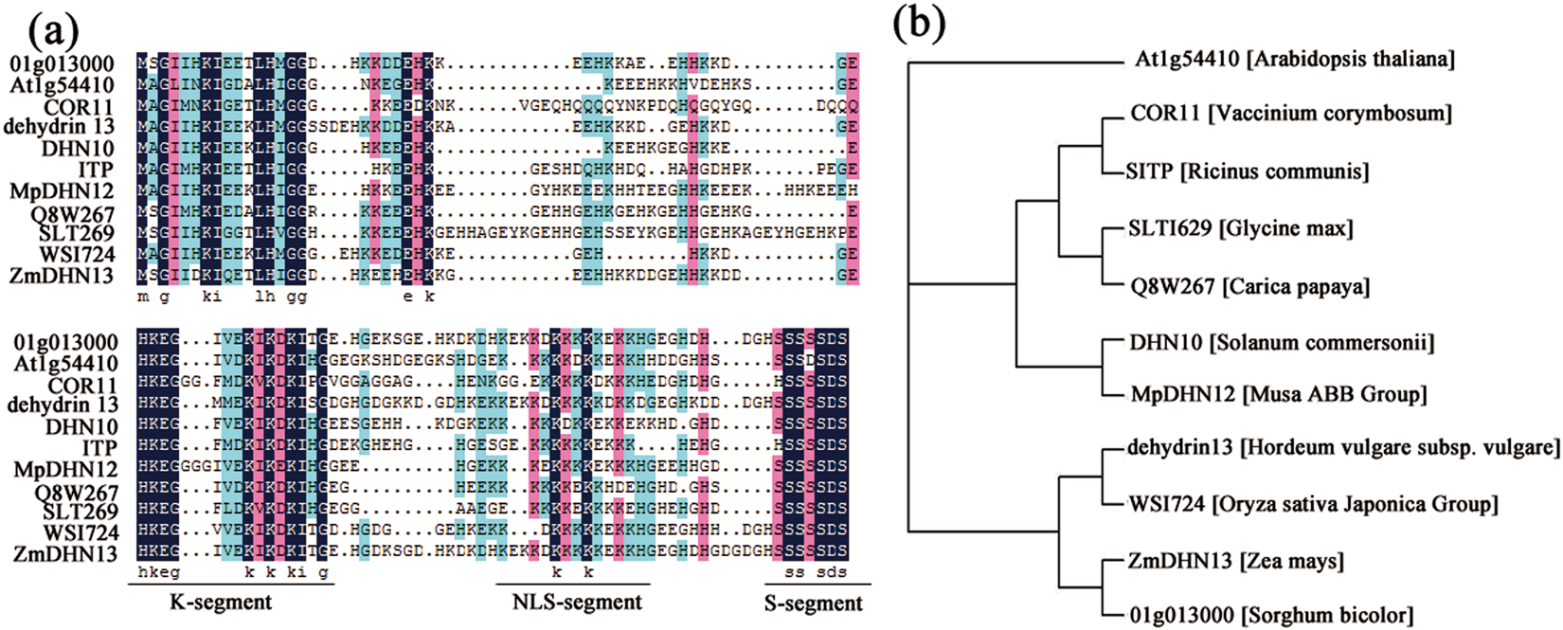
Sequence analysis of ZmDHN13 derived from maize and multiple sequence alignment and analysis of KS-type dehydrins from several plant species. (**a**) Multiple sequence alignment of ZmDHN13 with other KS-type dehydrin proteins. (**b**) Phylogenetic relationship of ZmDHN13 with other closely related LEA proteins. The unrooted dendrogram was constructed with the Tree View tool using the maximum likelihood method based on a complete protein sequence alignment of LEA proteins from other species.

The *ZmDHN13* (NP_001150115.1) cDNA has an open reading frame of 324 bp that encodes a protein of 107 amino acid residues with an estimated molecular mass of 12 kD and a pI of 6.33 (Fig. 1). The ZmDHN13 protein is rich in His (15%), Gly (14%), Lys (23.4%), and Glu (14%). According to the analysis of the conserved domains, ZmDHN13 is a KS-dehydrin. ZmDHN13 possesses a single chain of characteristic Ser residues, an S-segment in the C terminus, one K-segment and one NLS-segment. The ZmDHN13 protein displays a diverse homology with other KS-type dehydrins, and its motifs broadly match similar segments in related dehydrins, indicating a close evolutionary relationship between these proteins (Fig. 2).

### Accumulation of the *ZmDHN13* transcript under different stress treatments

**Figure 3:**
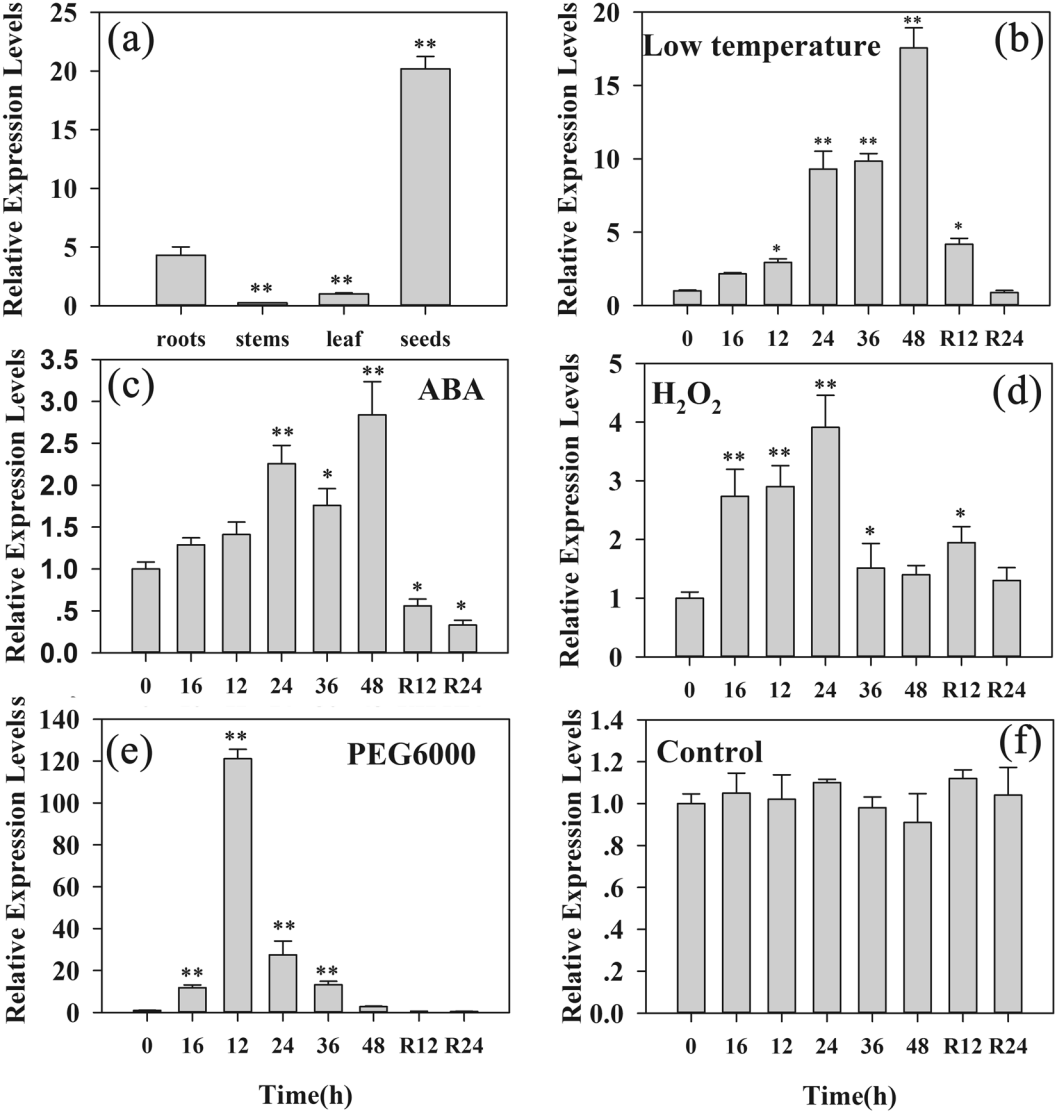
Transcript accumulation analysis of *ZmDHN13*. (**a**) Tissue-specific transcript accumulation of *ZmDHN13* under non-stress conditions; total RNA was isolated from the leaves, stems, roots and seeds. The maize seedlings were treated with 4 °C (**b**), 100 μM ABA (**c**), 20 mM H_2_O_2_ (**d**), 20% PEG6000 (w/v, **e**) or water (control, **f**). For the PEG6000, ABA and H_2_O_2_ treatments, maize plants were watered daily. Total RNA was isolated from roots (**b**) at the indicated times after the treatments. R represents removal of the treatments.

To determine the transcript patterns of *ZmDHN13*, quantitative real-time reverse transcription-PCR (qRT-PCR) was performed using RNA from stressed and non-stressed *Zea mays*. Under the 20% PEG treatment, the transcript of *ZmDHN13* in the roots peaked at 12 h but then decreased to its normal level. ABA or cold treatment caused the transcript of *ZmDHN13* in the roots to peak at 48 h but then decreased slowly to pre-induction levels. H_2_O_2_ caused a marked increase in the transcript level in the roots at 24 h, followed by a gradual reduction to the untreated levels (Fig. 3). The results demonstrated that the transcript accumulation of *ZmDHN13* could be induced by high osmosis, low temperature, H_2_O_2_ and ABA.

### Influence of different segments on the subcellular localization of *ZmDHN13*

**Figure 4:**
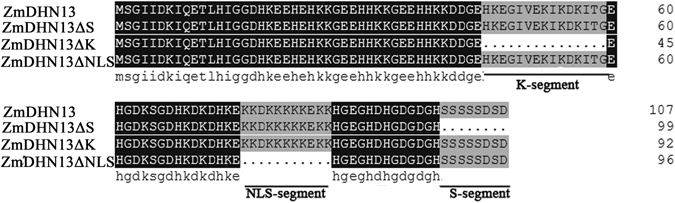
Comparison of the amino acid sequences of ZmDHN13 and the deletion mutants (ZmDHN13ΔS, ZmDHN13ΔK, and ZmDHN13ΔNLS). The conserved segments are shown as indicated.

To confirm the roles of the different segments in ZmDHN13, the K-, the NLS- and the S-segments were deleted from ZmDHN13. ZmDHN13 and the three mutant proteins (ZmDHN13ΔS, ZmDHN13ΔK and ZmDHN13ΔNLS) are shown in Fig. 4.

**Figure 5:**
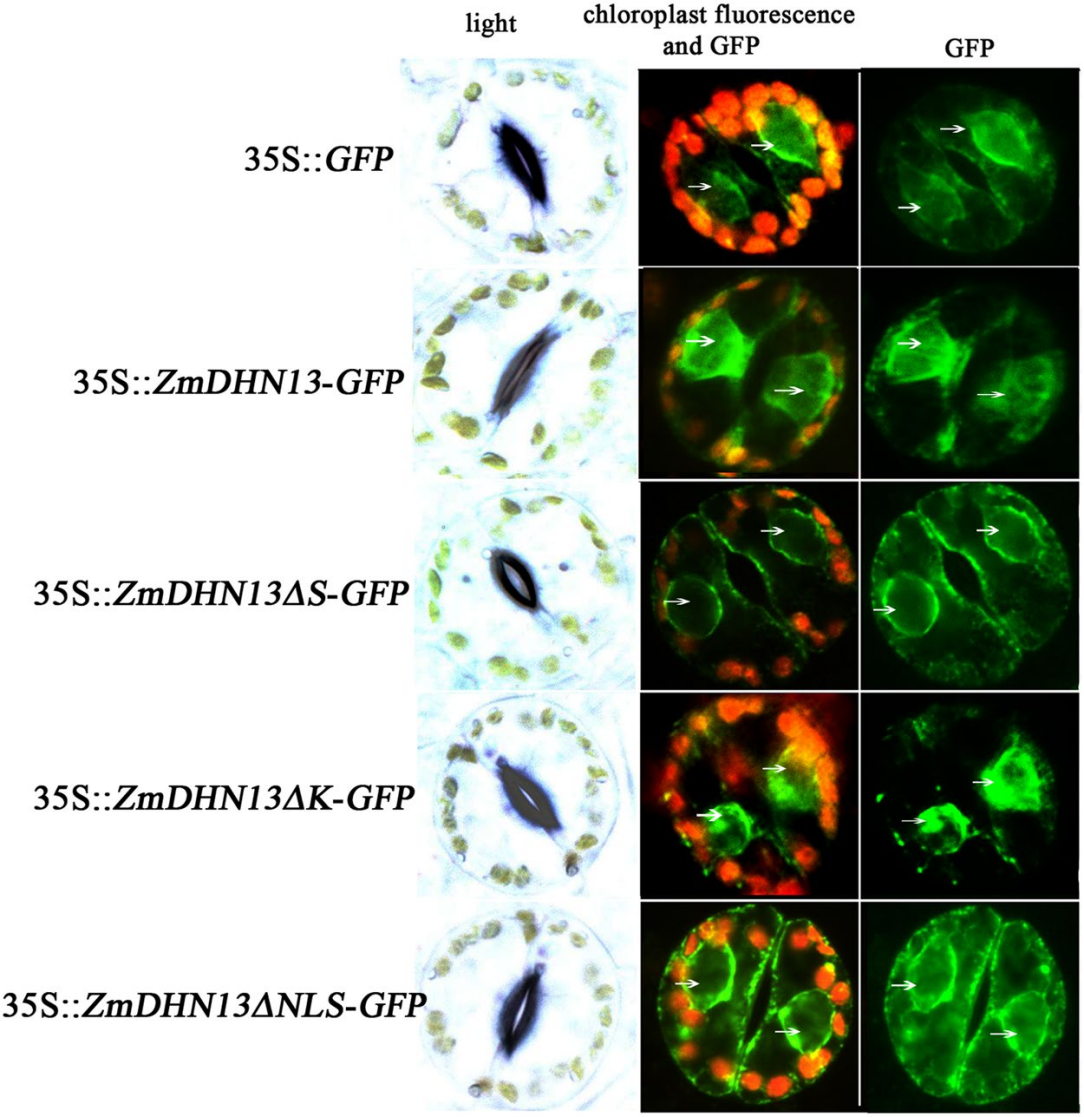
Subcellular localization of the GFP fusion proteins (ZmDHN13-GFP, ZmDHN13ΔS-GFP, ZmDHN13ΔK-GFP and ZmDHN13ΔNLS-GFP) in transgenic tobacco. The subcellular distribution of the GFP fusions and control GFP in the epidermal guard cells of transgenic tobacco using confocal laser scanning microscopy. Red is chloroplast fluorescence, and green is GFP. The arrows represent the nucleus.

Dehydrins can be localized to various cell compartments, including the cytosol, nucleus, plasma membrane, mitochondria and vacuole^19, 33–35^. Leaf epidermal cells of the transgenic tobacco plants expressing GFP fusion proteins (ZmDHN13:GFP, ZmDHN13ΔS:GFP, ZmDHN13ΔK:GFP and ZmDHN13ΔNLS:GFP) were examined using a Leica confocal laser scanning microscope. The fusion protein ZmDHN13:GFP and ZmDHN13ΔK:GFP were located in the nuclei and cytosol, but the signal of the fusion proteins mainly accumulated in the nucleus. In contrast, the fusion proteins ZmDHN13ΔS:GFP and ZmDHN13ΔNLS:GFP accumulated in nuclei and cytosol (Fig. 5). Thus, it is believed that the S-segment and the NLS-segment are essential for targeting of ZmDHN13 to the nucleus.

### The S- and NLS-segments play important roles in the phosphorylation

**Figure 6:**
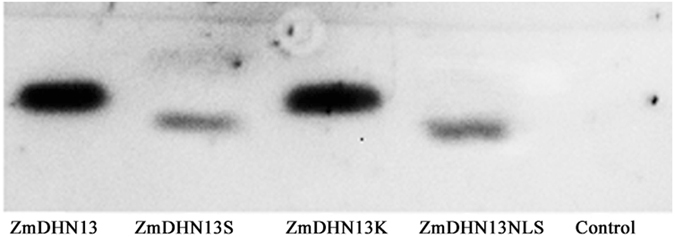
Phosphorylation analysis of ZmDHN13 and its deleted proteins. The recombinant proteins were phosphorylated by CKII. Lanes 1, 2, 3 and 4 indicate the phosphorylated proteins ZmDHN13, ZmDHN13ΔS, ZmDHN13ΔK and ZmDHN13ΔNLS, respectively. Lanes 5 indicates the non-phosphorylated protein ZmDHN13ΔNLS.

Casein kinase II (CKII) is a constitutively active serine/threonine protein kinase composed of two 44-kDa catalytic α-subunits and two 26-kDa regulatory β-subunits in a α_2_β_2_ configuration to form stable heterotetramers. CKII was used for the *in vitro* phosphorylation assays with the recombinant ZmDHN13 proteins and the deleted proteins. The proteins ZmDHN13 and ZmDHN13ΔK could be phosphorylated by CKII. However, the ability to phosphorylate was partly inhibited for the proteins ZmDHN13ΔS and ZmDHN13ΔNLS (Fig. 6). We performed yeast two-hybrid screening to identify whether the ZmDHN13 protein could interact with CKII (CKII-α1, CKII-α2, CKII-α1, CKII-β1, CKII-β2 and CKII-β3). However, there was no specific interaction with enzymes under control conditions (data not shown). These results demonstrated that the NLS-segment and the S-segment play important roles in the process of phosphorylation.

### The K-segment of ZmDHN13 is essential for the protective effect of LDH activity during *in vitro* oxidative stress

**Figure 7:**
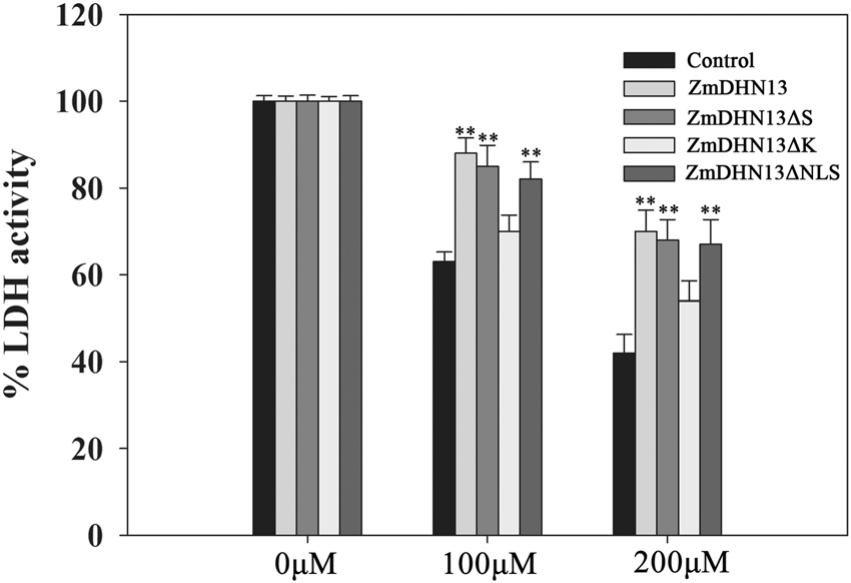
ZmDHN13 could Protect LDH activity during oxidation stress. Effects of oxidation on LDH activity after 100 μM or 200 μM H_2_O_2_ treatment. LDH activity was measured in the presence of 0.24 mg of ZmDHN13 protein or 0.24 mg of BSA protein (control).

Some studies have shown that dehydrins protect enzymatic activities (LDH, CS) during *in vitro* partial water limitation or freezing^36–38^. To obtain more detailed information, the rate of LDH inactivation during water loss was determined with or without the ZmDHN13 protein. When 100 or 200 μM H_2_O_2_ was added, LDH retained 70% of its initial activity when additional ZmDHN13 protein was added, whereas LDH (alone) lost approximately 60% of its initial activity. When the deleted proteins ZmDHN13ΔS and ZmDHN13ΔNLS were added, LDH also retained 68% of its initial activity when 100 or 200 μM H_2_O_2_ was added. However, LDH lost approximately 50% of its initial activity when the ZmDHN13ΔK proteins were added (Fig. 7). These results demonstrated that the ZmDHN13 protein could preserve LDH activity and that the K-segment was the core segment in the protective effect of LDH activity during oxidative stress.

### Overexpression of ZmDHN13 enhances transgenic tobacco tolerance to oxidative stress

To explain the biological function of ZmDHN13 in plants, transgenic tobacco plants overexpressing ZmDHN13 and deleted genes under the control of the CaMV 35S promoter were selected for further analysis. Independent transgenic lines were obtained by kanamycin-resistance selection and confirmed by genomic PCR. Three plants of each transgenic line were selected for further analysis; the transgenic lines displayed similar transcript levels (Supplementary Figure 1). The transgenic line containing the pBI121-GFP empty vector was used as a control.

**Figure 8:**
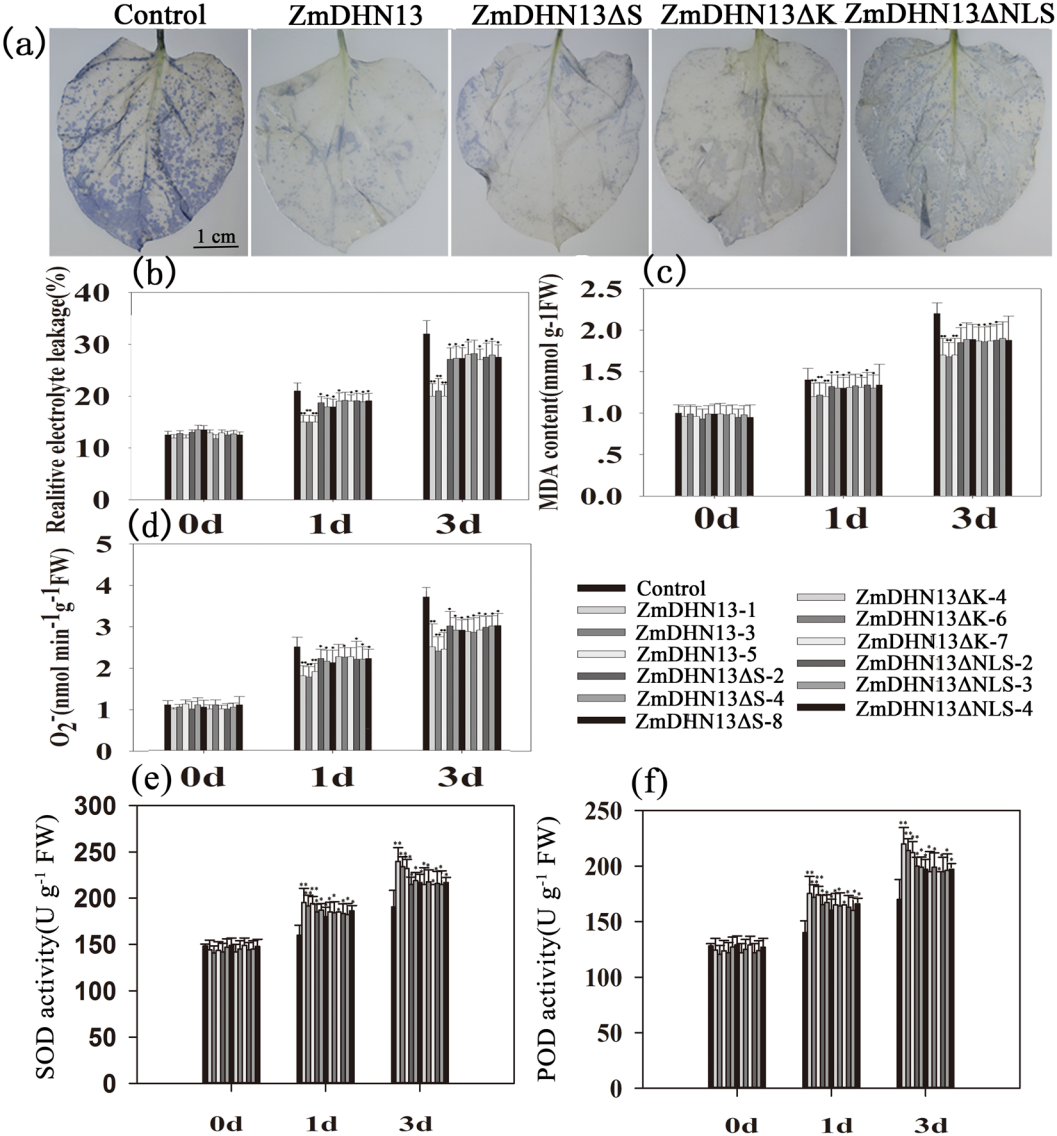
Assay of oxidative stress tolerance of transgenic tobacco plants. Transgenic and control tobacco plants were grown at a normal temperature (25 °C) for 6 weeks before use. (**a**) The leaves of transgenic and control tobacco plants were sprayed with 20 mM H_2_O_2_. *In situ* detection of O_2_^−^ by NBT staining of control and transgenic leaves was performed after 12 h. The electrolyte leakage (**b**), MDA (**c**), O_2_^−^ (**d**), SOD activity (**e**) and POD activity (**f**) in transgenic and control tobacco plants were measured at the indicated times after treatment with 20 mM H_2_O_2_. The experiment was repeated three times with similar results obtained. The statistical significance of the difference was confirmed by Student’s t–test. The data of the control plant and the transgenic plant were tested for significance differences at each respective time point. *P < 0.05. **P < 0.01.

Environmental stresses result in the accumulation of ROS in plants. We evaluated the accumulation of ROS in transgenic and control seedlings under oxidative stress. After treatment, the ZmDHN13-overexpressing lines showed much less accumulation of the superoxide radical (O_2_^−^) relative to that of the control lines. The amounts of relative electrolyte leakage and MDA in the control plants were higher than those in the transgenic lines after H_2_O_2_ treatments (Fig. 8). The results indicated that the overexpression of ZmDHN13 in transgenic tobacco could enhance tolerance to oxidative stress.

To further explain the biological function of the conserved ZmDHN13segments in plants, *ZmDHN13ΔK*, *ZmDHN13ΔS* and *ZmDHN13ΔNLS* were overexpressed in transgenic tobacco under the control of the CaMV 35S promoter. After treatment, the three transgenic tobacco lines (*ZmDHN13ΔK*, *ZmDHN13ΔS* and *ZmDHN13ΔNLS*) also displayed a higher tolerance than that of the control plants, but the protective roles were lower than those of the *ZmDHN13* transgenic tobacco plants (Fig. 8). According to these results, we conclude that the overexpression of *ZmDHN13* enhances the transgenic tobacco tolerance to oxidative stress, which partly depends on the three conserved segments.

## Discussion

Dehydrins are classified into five subclasses. By definition, a dehydrin must contain at least one copy of the K-segment. KS-type dehydrins have the simplest structure and contained one K-segment, one NLS-segment and one S-segment. This fact made it possible to more easily identify the biochemical and physiological functions of the three conserved segments using the mutant.

Dehydrins can retain water molecules and prevent the crystallization of cellular components under water deficit that results from drought, high salt and freezing stresses. *Arabidopsis thaliana AtHIRD11* and the barley KS-type dehydrin *dhn13* are constitutively expressed^30, 39^. The expression of *ZmDHN13* is also constitutive but can be induced by various abiotic stresses, including high-osmotic, low temperature, and oxidative stress, as well as ABA application. These results similar to those involving *DHN1*0 in *Solanum tuberosum* ^22^.

Phosphorylation is a significant feature of dehydrins containing the S-segment. The maize dehydrin Rab17 can be phosphorylated by protein kinase CKII^24, 40^. Here, ZmDHN13 could also be phosphorylated by CKII, depending on the S- and NLS-segments. CKIIα contains the region (KKKKIKR) immediately preceding the conserved glutamic acid of region III. It has been postulated that this region may be interact with the acidic residues of protein or peptide substrates^41^. Many studies have demonstrated that dehydrin can bind with phospholipids. The interaction requires basic amino acids such as arginine and lysine in the binding domain of dehydrins. An electrostatic nature of the interaction has been postulated^14, 15, 42, 43^. The NLS-segment of ZmDHN13 contained a stretch of basic amino acid residues (KKDKKKKKEKK). Deleting the NLS-segment can affect net charge of the proteins. Therefore, we speculate that the NLS-segment might mediate the interaction between the CKII and the protein ZmDHN13 by electrostatic repulsion. However, we found the ZmDHN13 protein did not interact with the α or β subunit of CKII. We speculated that the ZmDHN13 protein might interact with domains that consist of two or more subunits of CKII, but this fascinating possibility awaits further investigation.

Previous studies have demonstrated that KS-type dehydrins are located in the nucleus and cytoplasm^22, 30^. In this study, the S- and the NLS-segments rather than the K-segment were the core sequences required for the nuclear location of ZmDHN13. The S-segment of the dehydrin Rab17 is the core sequence for phosphorylation and nuclear localization^44, 45^. Although there were some differences in the amino acid sequence of the S-segment, the segment played the same roles as did that of Rab17. A stretch of basic amino acid residues (MGGRRKKP) in the Rab17 protein located in the central part of the protein, resembling the SV40 type NLS, fused to GUS could partially deliver the fusion protein to the nucleus^24, 46^. In the present study, the NLS-segment of ZmDHN13 also contained a stretch of basic amino acid residues (KKDKKKKKEKK) that resembled the NLS of the dehydrin Rab17. Recently, studies have also demonstrated that the localization of the cactus dehydrin OpsDHN1 is mediated by the histidine-rich motif (HHKEQEEEQEDKQKDHHHHHHDEED) and the S-segment^47^. It is important to mention that histidine (H), lysine (K) and arginine (R) are the basic amino acids. Therefore, it is reasonable to speculate that basic amino acid-rich segments and the S-segments play similar roles in nuclear localization.

Protein denaturation is one of the most common physiological phenomena that occurrs in plant cells exposed to stress. Many studies have shown that dehydrins can protect enzyme activities (LDH, CS) against damage caused by various stresses^36, 37^. The K-segment of the wheat dehydrin DHN-5 is essential for the protection the LDH and β-glucosidase activities *in vitro* ^39^. The dehydrins ERD10 and RcDhn5 effectively prevented LDH inactivation during freeze-thaw cycles, and the K-segments of dehydrins were essential to this activity. The K-segment can form amphipathic helicity, which may be related to its function^37^. In the present study, ZmDHN13 could protect enzyme (LDH and antioxidant enzymes) activity from the damage caused by oxidative stress. Additional studies indicated that the K-segment was essential to the protection of enzyme activity during oxidative stress conditions.

Metals such as copper and zinc are essential for gene expression and metabolic processes involved in plant growth; however, reactive transition metals are released from enzymes and organelles under environmental stresses. Free metals are the source of ROS generation^48–52^. Many dehydrins stabilize transition metal ions by binding them^53, 54^. The *Ricinus* KS-type dehydrin ITP was the first member of the LEA protein family reported to be involved in the long distance transport of micronutrients^55^. The *Arabidopsis thaliana* KS-type dehydrin AtHIRD11 can bind metal ions and reduce ROS generation from Cu. It has also been proposed that the histidine content and the length of the peptides are fundamental factors that influence the strength of the ROS reduction by KS-type dehydrins^56^.

Lipid peroxidation results from reactive oxygen species (ROS) that are generated in stressed plants^32, 46^. The dehydrin AtHIRD11 has ROS-reducing activity, which can be determined by the histidine contents and the length of the peptide^56^. Among the amino acid residues of ZmDHN13, Gly, His, and Lys account for 14%, 15%, and 23.4%, respectively. These three amino acids are targets for hydroxyl-radical mediated protein oxidation; they are most severely degraded by hydroxyl radicals^57^. In this study, the overexpression of *ZmDHN13* enhanced transgenic tobacco tolerance to oxidative stress. The *ZmDHN13ΔS*, *ZmDHN13ΔK* and *ZmDHN13ΔNLS* transgenic tobacco also displayed a higher tolerance than control tobacco under oxidative stress, but these roles were weaker in *ZmDHN13ΔS*, *ZmDHN13ΔK* and *ZmDHN13ΔNLS* transgenic tobacco than in *ZmDHN13* transgenic tobacco. The K-, S- and NLS-segments influenced the protein subcellular localization, which also plays an important roles in the response to environmental stresses *in vivo*. According to these results, we conclude that the ZmDHN13 protein could enhance transgenic tobacco tolerance to oxidative stress through diverse mechanisms and that the three conserved segments exhibit a cooperative effect in response to various stresses *in vivo*.

Phosphorylation plays an important role in plant signal transduction and stress responses. In the present study, the roles of the S- and NLS-segments were nearly identical to those of the K-segment in response to oxidative stress in plants. The K-segment of ZmDHN13 most likely forms an α-helix that interacts with membranes or proteins, modulating their phase properties and conformational transitions. The S- and the NLS-segments could influence the phosphorylation and localization of the protein, which mainly affect its roles *in vivo*.

In summary, our data suggest that the basic amino acid-rich segment (the NLS-segment) and the S-segment play similar roles in nuclear localization of the different dehydrins types. The KS-type dehydrins might have a complex response mechanism under various environmental stresses, and the three conserved segments exhibit a cooperative effect in response to environmental stresses *in vivo*.

## Electronic supplementary material

Supplementary Dataset
