# Supplementary material for: Functional characterization of KS-type dehydrin ZmDHN13 and its related conserved domains under oxidative stress
Source: Sci Rep. 2017 Aug 4;7:7361. doi: 10.1038/s41598-017-07852-y (PMC5544677; doi:10.1038/s41598-017-07852-y)
Supplement: Supplementary file 1 — Supplementary Dataset [file 41598_2017_7852_MOESM1_ESM.doc]

**Title:** Functional characterization of KS-type dehydrin ZmDHN13 and its related conserved domains under oxidative stress

**Author:** Yang Liu**,** Li Wang, Tianpeng, Zhang, Xinghong Yang, Dequan Li,

**Supplementary Figure1**

**
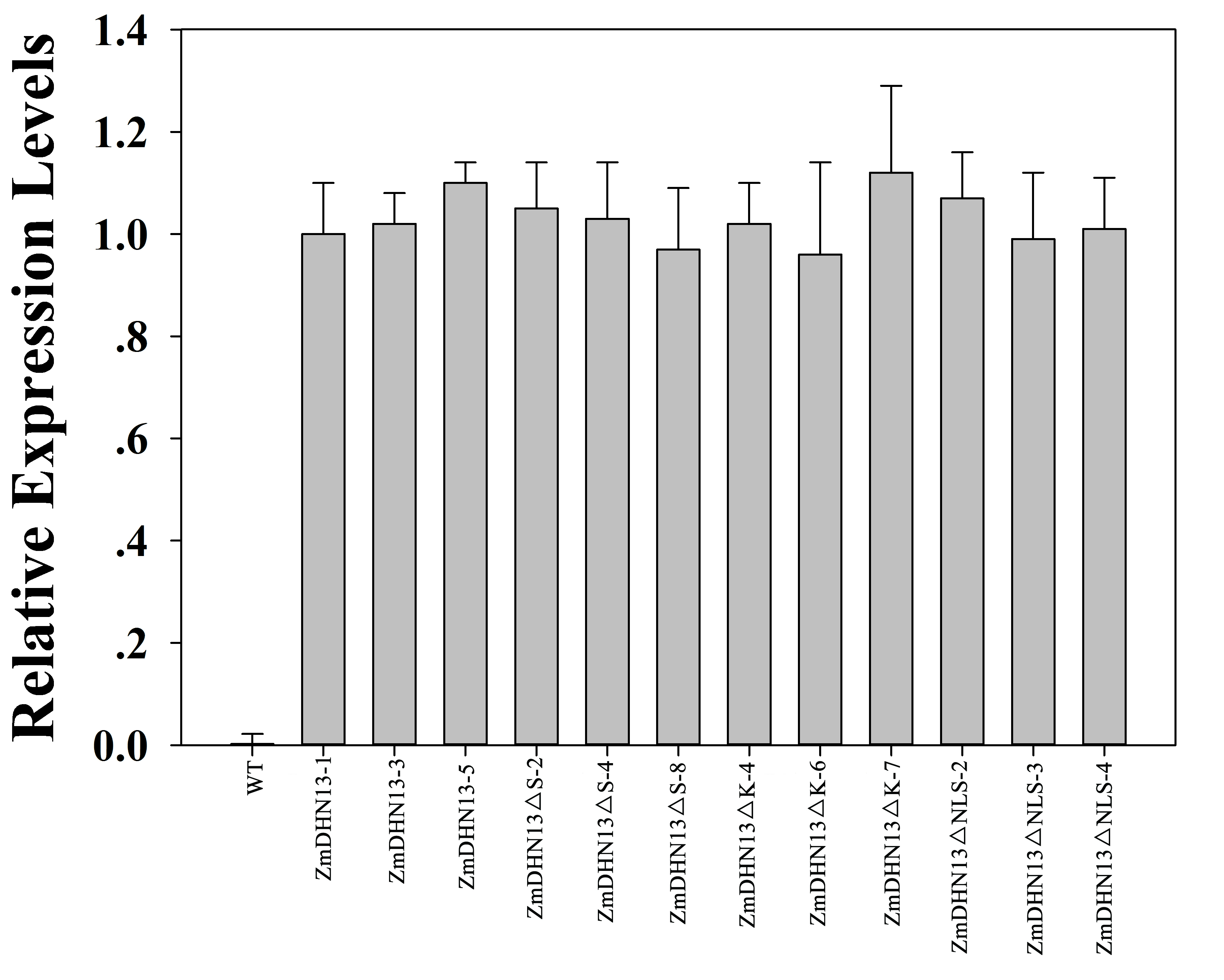
**

**Figure legends:** *ZmDHN13*, *ZmDHN13ΔS*, *ZmDHN13ΔK* and *ZmDHN13ΔNLS* transcript accumulation in transgenic tobacco plants. The expression is relative to that of the WT. Total RNA was isolated from leaf samples collected from T2 tobacco plants under normal conditions.

**Supplementary Method1** Cloning of different Segment Deletion genes *ZmDHN13ΔK* and *ZmDHN13ΔNLS*

*ZmDHN13ΔK* and *ZmDHN13ΔNLS* were generated using overlap extension PCR as described earlier with modifications (Heckman and Pease, 2007). PCR overlap extension can create specific nucleotide mutations, the principle was shown below:


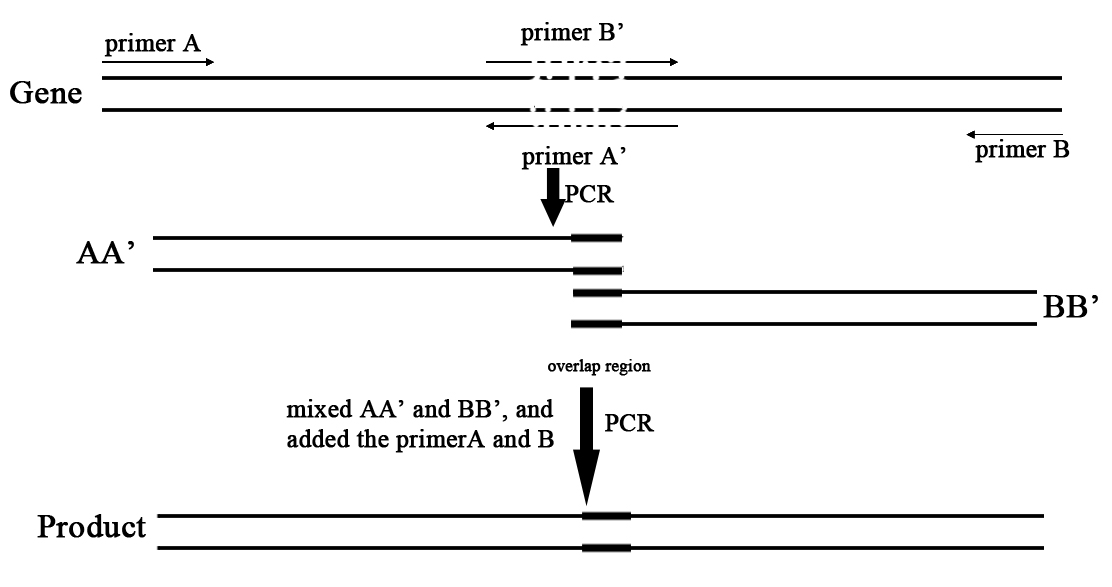


**Figure legends:** The dashed line indicated the deleted sequence. The broad line indicated the overlap sequence. The figure refers to the article (Heckman and Pease, 2007).

The up-region sequence of *ZmDHN13ΔK* was amplified with primers (forward GGATCCAGAGAAGTAGCCACAAGCATG *Bam*HI site underlined and reverse CTTGTCGCCGTGCTCCTCCCCGTCGTCCTT). The down-region sequence of *ZmDHN13ΔK* was amplified with primers (forward AAGGACGACGGGGAGGAGCACGGCGACAAG and reverse GAGCTCACAACAATCTTGGCGAGT *Sac*I site underlined).

The up-region sequence of *ZmDHN13ΔNLS* was amplified with primers (forward GGATCCAGAGAAGTAGCCACAAGCATG *Bam*HI site underlined and reverse ATGGCCCTCCCCGTGCTCCTTATGGTCTTT). The down-region sequence of *ZmDHN13ΔK* was amplified with primers (forward AAAGACCATAAGGAGCACGGGGAGGGCCAT and reverse GAGCTCACAACAATCTTGGCGAGT *Sac*I site underlined).

The up-region sequence and the up-region sequence were isolated by 1% (w/v) agarose gel, Purify the PCR products using Gel Extraction kit. Products up-region sequence and the up-region sequence as template DNA for this PCR.

| Component | Final concentration |
| --- | --- |
| 10×PCR buffer with MgCl2 | 1× |
| 10×dNTPs | 1× |
| Up-region sequence products | 100 ng |
| Down-region sequence products | 100 ng |
| Taq polymerase | 5U |
| Water | to 50 μl |

Place tubes in PCR machine and run using the program below:

| Cycle number | Denature | Denature | Anneal | Extend |
| --- | --- | --- | --- | --- |
| 1 | 94℃, 5min |  |  |  |
| 2-7 |  | 94℃, 1 min | 42-55℃, 1 min | 72℃, 1 min |

After reaction, the primers (forward GGATCCAGAGAAGTAGCCACAAGCATG *Bam*HI site underlined and reverse GAGCTCACAACAATCTTGGCGAGT *Sac*I site underlined) were added and run using the program below:

| Cycle number | Denature | Denature | Anneal | Extend | Hold |
| --- | --- | --- | --- | --- | --- |
| 1 | 94℃, 5min |  |  |  |  |
| 2-35 |  | 94℃, 1 min | 42-55℃, 1 min | 72℃, 1 min |  |
| 3 |  |  |  | 72℃, 10 min |  |
| 4 |  |  |  |  | 4℃,10min |

The PCR product was cloned into the PMD18-T vector and sequenced.

**Supplementary Method2** Determination of superoxide radical (O_2_^–^)

O_2_^–^ was measured as described by Jiang and Zhang (2001). 1g of frozen leaf segments was homogenized with 3 ml of 65 mM potassium phosphate buffer (pH 7.8) and centrifuged at 5,000×g for 10 min. The incubation mixture contained 0.9 ml of 65 mM phosphate buffer (pH 7.8), 0.1 ml of 10 mM hydroxylamine hydrochloride, and 1 ml of the supernatant. After incubation at 25℃ for 20 min, sulfanilamide (17 mM) and naphthylamine (7 mM) were added to the incubation mixture. After reaction at 25℃ for 20 min, ethyl ether in the same volume was added and centrifuged at 1,500×g for 5 min. The absorbance in the aqueous solution was read at 530 nm. A standard curve with NO_2_^–^ was used to calculate the production rate of O_2_^–^ from the chemical reaction of O_2_^–^ and hydroxylamine.

**Supplementary Method3** Determination of the MDA

The MDA content was determined using the thiobarbituric acid (TBA) reaction. Leaf samples (0.5 g) of tobacco plants were homogenized in 5 ml of 10% (w/v) trichloroacetic acid (TCA) and centrifuged at 12,000 rpm for 10 min at 4℃. Subsequently, 2 ml of 0.6 % (w/v) thiobarbituric acid (TBA) in 10% TCA (w/v) was added to 2 ml of the supernatant. The mixture was heated in boiling water for 15 min and then quickly cooled in an ice bath. Following centrifugation at 12,000 rpm for 10 min at 4℃, the absorbance of the supernatant at 450, 532 and 600 nm was determined spectrophotometrically. The concentration of malondialdehyde was calculated by the following equation: MDA content (μmol l^-1^) = 6.45 (A_532_–A_600_)–0.56 A_450_.

**References**

Heckman, K.L., Pease, L.R. (2007) Gene splicing and mutagenesis by PCR-driven overlap extension. Nature protocols 2, 924–932

Jiang M, Zhang J. (2001) Effect of abscisic acid on active oxygen species, antioxidative defense system and oxidative damage in leaves of maize seedlings. Plant Cell Physiol. 42: 1265–1273
